# Fault-Tolerant Control for Quadcopters Under Actuator and Sensor Faults

**DOI:** 10.3390/s24227299

**Published:** 2024-11-15

**Authors:** Kenji Fabiano Ávila Okada, Aniel Silva Morais, Laura Ribeiro, Caio Meira Amaral da Luz, Fernando Lessa Tofoli, Gabriela Vieira Lima, Luís Cláudio Oliveira Lopes

**Affiliations:** 1 Faculty of Electrical Engineering, Federal University of Uberlândia, Uberlândia 38408-100, Brazil; kenji_okada@ufu.br (K.F.Á.O.); laurar@ufu.br (L.R.); gabriela.lima@ufu.br (G.V.L.); 2 Department of Electrical Engineering, Federal University of São João del-Rei, São João del-Rei 36307-352, Brazil; caiomeiramaral@hotmail.com (C.M.A.d.L.); fernandolessa@ufsj.edu.br (F.L.T.); 3 Faculty of Chemical Engineering, Federal University of Uberlândia, Uberlândia 38408-100, Brazil; lcol@ufu.br

**Keywords:** fault detection and diagnosis, fault-tolerant control, Kalman filter, quadcopters, unmanned aerial vehicles

## Abstract

Fault detection and diagnosis (FDD) methods and fault-tolerant control (FTC) have been the focus of intensive research across various fields to ensure safe operation, reduce costs, and optimize maintenance tasks. Unmanned aerial vehicles (UAVs), particularly quadcopters or quadrotors, are often prone to faults in sensors and actuators due to their complex dynamics and exposure to various external uncertainties. In this context, this work implements different FDD approaches based on the Kalman filter (KF) for fault estimation to achieve FTC of the quadcopter, considering different faults with nonlinear behaviors and the possibility of simultaneous occurrences in actuators and sensors. Three KF approaches are considered in the analysis: linear KF, extended KF (EKF), and unscented KF (UKF), along with three-stage and adaptive variations of the KF. FDD methods, especially the adaptive filter, could enhance fault estimation performance in the scenarios considered. This led to a significant improvement in the safety and reliability of the quadcopter through the FTC architecture, as the system, which previously became unstable in the presence of faults, could maintain stable operation when subjected to uncertainties.

## 1. Introduction

### 1.1. Problem Statement

Unmanned aerial vehicles (UAVs) have consistently attracted global investment due to the growing demand for these devices in solving various problems across different sectors, such as object detection, tracking and surveillance, data collection, route planning, navigation, collision prevention, coordination, environmental and energy monitoring, and agriculture [1]. Among UAVs, quadcopters or quadrotors stand out for their vertical takeoff and landing capabilities, hovering simple mechanical structure, single moving part per actuator, low cost, and easy maintenance [2]. In this context, continuous technological improvement of such aircraft is necessary to enhance efficiency and safety, ensuring reliability, as accidents can result from failures in actuators, structures, or sensors [3].

Unfortunately, failures in UAVs are inevitable and often lead to irreparable financial losses in practical scenarios. Failures refer to any deviation of a system’s characteristic from acceptable conditions, potentially degrading its ability to perform its function [4]. Failures can manifest as abrupt, intermittent, or noisy issues, among others. Preventive measures to enhance safety and reliability include process monitoring, system reconfiguration, regular maintenance, and installation of secure components [5]. Studies indicate that transmission, communication, battery, and navigation failures occur most frequently in UAVs. Thus, fault-tolerant control (FTC) and stability mechanisms are crucial in this field [6].

Given the above, fault detection and diagnosis (FDD) and FTC strategies have provided significant solutions to these challenges [7]. FDD focuses on extracting detailed information about failures, such as detection, location, and magnitude, while FTC involves control structures designed to mitigate the effects of failures on system performance. Various FDD and FTC approaches have been applied to UAVs, particularly quadcopters. However, there is still a need for robust and efficient strategies that address the specific properties of these aircraft and diverse failure scenarios [7].

### 1.2. Literature Review

FTC methods enable the design of control systems that can mitigate or suppress the harmful effects of process failures, either actively or passively. FDD strategies are crucial to monitoring systems, representing the combination of detection and diagnosis, using signal analysis methods, data-driven approaches, and model-based techniques [8]. Passive FTC is defined in [9] as a static approach, where a single controller handles faults. In contrast, active FTC is more dynamic, adapting to faults as they occur through the information generated by the FDD system. According to [10], active FTC is commonly applied in unmanned aerial systems (UASs) and quadrotors.

FDD applications in UAVs can vary depending on the type of fault and the solution adopted. One of the main challenges associated with signal-based methods is noise, which can be generated by the sensors or other external sources [11]. Fault detection relying on time and frequency domain signal analysis is proposed in [12], while the discrete Fourier transform is applied to the sensor signals in [13]. Both studies achieved satisfactory results and managed to mitigate the noise effects on the signal. However, they are limited to fault detection only and do not account for situations involving multiple or simultaneous faults.

Instead of relying solely on signal analysis techniques for fault detection in UAVs, data-driven FDD methods have gained popularity due to the increasing complexity of systems and advances in data processing and storage capabilities. These methods are divided into two groups: those based on statistical analysis and those based on artificial intelligence, such as artificial neural networks (ANNs) [14]. However, the computational burden can be high enough to make the implementation of these techniques unfeasible [15]. Additionally, these methods are limited to the qualitative classification of faults for diagnostics and require considerable information—often difficult to obtain experimentally—to correctly detect and identify only specific types of faults in the quadrotor, as outlined in [16].

Another approach investigated in the literature is based on hybrid structures combining two or more FDD systems. The method used in [17] proposes an adaptive neural network approach with online weight calculation based on the extended Kalman filter (EKF) for fault detection and diagnosis in the elevators, rudder, and ailerons of a fixed-wing UAV. Other studies combine the radial basis function neural network (RBFNN) with different algorithms [18,19]. A hybrid structure is implemented in [20], consisting of sensor fault diagnosis through deep learning based on convolutional neural networks (CNNs). Using the hybrid structure, the authors improved certain features of the FDD systems compared to other methods. However, their solutions addressed faults in either actuators or sensors, but not in both simultaneously.

Model-based methods are better recommended for FDD because they allow for diagnosis based on fault estimation, enabling isolation and determination of the time of occurrence, magnitude, and location of faults. However, model uncertainties can affect its performance [21]. In this case, diagnosis occurs when model parameters are related to a system component, when the simultaneous analysis of several generated residuals allows for fault localization, and when faults are estimated using filters or observers [22]. However, model uncertainties can affect its performance, and obtaining these models becomes even more complex with large-scale systems and operations involving a significant number of variables [23].

In different cases relying on model-based methods, other aspects of FDD and FTC have been considered, such as the increased computational burden due to the inclusion of faults and disturbances in the system states, and the uncertainty in the dynamics of these signals. Actuator fault estimation in quadcopters is addressed in [24] using the adaptive three-stage Kalman filter (ATsKF), which is designed without requiring precise assumptions about the stochastic characteristics of faults and external disturbances. The filter gain adapts according to the actual value of the covariance matrices, and the filter’s three-stage structure reduces the computational burden, though the approach assumes a linear model.

Conversely, the three-stage unscented Kalman filter (TsUKF) and robust three-stage unscented Kalman filter (RTsUKF) were used in [25] for estimating faults and external disturbances, taking into account the system’s nonlinear model through the structure of the unscented Kalman filter (UKF). An RTsUKF was also designed in [26,27] considering various faults and unknown inputs, and it was also applied to a nonlinear system. However, such KFs were not applied to quadrotors.

Overall, it is reasonable to state that FDD relies on system monitoring, involving fault detection and determining its characteristics. Conversely, FTC can mitigate or suppress the fault influence on the processing while requiring FDD in many cases [28]. Table 1 summarizes the characteristics of existing methods. Some solutions available in the literature focus on fault detection and/or isolation, while others not only address these issues but can also ensure continuous system operation and mitigate the harmful effects of faults. Signal-based methods are often used for component monitoring and are primarily limited to fault detection, while data-driven methods can provide fault diagnosis but are restricted to classification and face challenges imposed by the nature of neural networks. Model-based FDD techniques prove to be more efficient in terms of the amount of information provided, allowing for better performance of the FTC system [29]. They are developed to address the non-linearities present in flight dynamics, external disturbances such as wind, model uncertainties, noise, and computational burden [30]. However, accounting for all these features in the design of a model-based structure remains a challenge.

### 1.3. Contributions and Organization

Given the above, this work aims to design and implement different model-based FDD approaches for a quadcopter and to develop an FTC system that addresses sensor and actuator faults. The main contributions of the study are as follows:

- providing a comprehensive description of the actuator and sensor faults considered in the analysis;
- implementing an adaptive version of the TsUKF, as proposed by [25], and assessing its application in quadrotors to address the main points highlighted in Section 1.2: the nonlinearities of the model, the lack of information regarding fault behavior, and the computational burden caused by the increase in system states due to the inclusion of fault estimations;
- applying linear, extended, and unscented Kalman filter (KF) approaches, including three-stage and adaptive versions for fault estimation;
- performance analysis in different flight scenarios, considering various fault behaviors and simultaneous fault occurrences;
- development of an FTC system for a quadcopter, integrating the results from the FDD system;
- performance comparison of the control structure with and without the FTC system under distinct scenarios, considering external disturbances, such as wind.

The remainder of this work is organized as follows. Section 2 details the dynamic modeling of a quadcopter. Section 3 addresses the FTC system, while Section 4 discusses the results obtained from various approaches. Section 5 concludes the study and points to future investigations.

## 2. Dynamic Modeling of the Quadcopter

**Figure 1:**
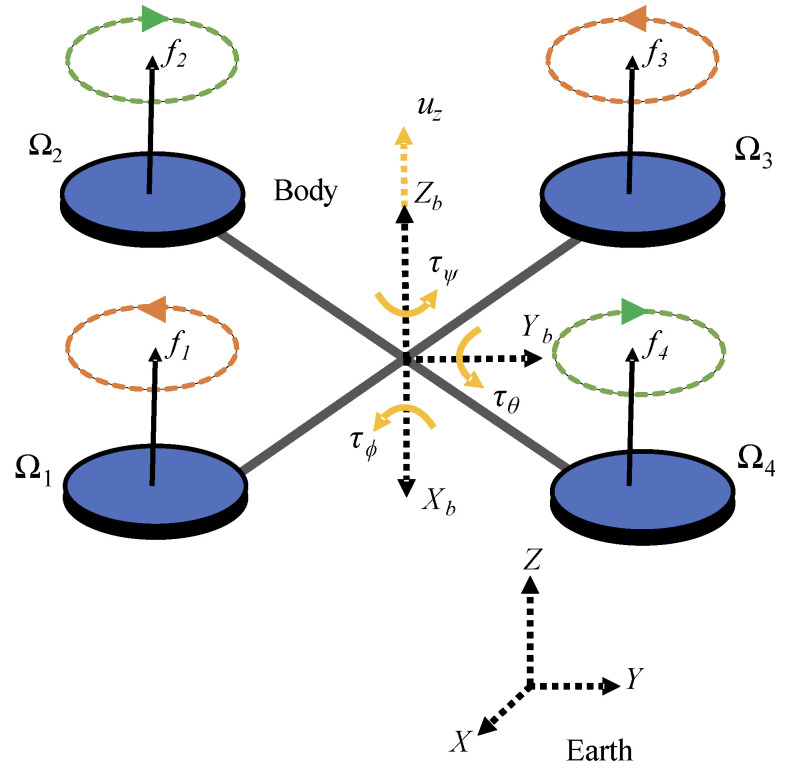
Quadcopter structure and variables.

For this application, it is essential to understand the modeling of the quadcopter. Its mathematical model can be obtained using the Newton-Euler formulation, but the whole procedure will not be presented here for simplicity [31]. The quadcopter is shown in Figure 1, which includes the inertial coordinate system (*X*, *Y*, *Z*) fixed to the Earth, and the body coordinate system (*X_b_*, *Y_b_*, *Z_b_*), which moves according to the aircraft’s orientation.

The forces (*f*_1_, *f*_2_, *f*_3_, *f*_4_) and the torques ($\tau_{1}$, $\tau_{2}$, $\tau_{3}$, $\tau_{4}$) generated by each propeller vary according to the angular velocity (*Ω*_1_, *Ω*_2_, *Ω*_3_, *Ω*_4_) of each motor and are related to the body-fixed coordinate system. To balance the counter-torque present in the motors, which is responsible for generating the yaw torque ($\tau_{\psi }$) and consequently the rotation of the quadcopter around the *Z_b_* axis, one pair of motors rotates clockwise while the other pair rotates counterclockwise.

The pitch torque $\tau_{\theta }$, responsible for rotation around the *Y_b_* axis, results from the difference between the forces (*f*_1_, *f*_4_) and (*f*_2_, *f*_3_), allowing the quadcopter to move along the *X_b_* axis. As for the roll torque ($\tau_{\phi }$), the difference lies between the forces (*f*_1_, *f*_2_) and (*f*_3_, *f*_4_) which enables rotating around the *X_b_* axis and a corresponding movement along the *Y_b_* axis according to (1). The parameter *l* represents the distance between the quadcopter’s center of mass and each motor.

(1) $$\tau_{B}=\left[\begin{array}{c} \tau_{\phi }\\ \tau_{\theta }\\ \tau_{\psi } \end{array}\right]=\left[\begin{array}{ccc} -\frac{bl}{\sqrt{2}} & -\frac{bl}{\sqrt{2}} & \begin{array}{cc} \frac{bl}{\sqrt{2}} & \frac{bl}{\sqrt{2}} \end{array}\\ -\frac{bl}{\sqrt{2}} & \frac{bl}{\sqrt{2}} & \begin{array}{cc} \frac{bl}{\sqrt{2}} & -\frac{bl}{\sqrt{2}} \end{array}\\ d & -d & \begin{array}{cc} d & -d \end{array} \end{array}\right]\left[\begin{array}{c} \Omega_{1}^{2}\\ \Omega_{2}^{2}\\ \begin{array}{c} \Omega_{3}^{2}\\ \Omega_{4}^{2} \end{array} \end{array}\right]=T_{\tau }\left[\begin{array}{c} \Omega_{1}^{2}\\ \Omega_{2}^{2}\\ \begin{array}{c} \Omega_{3}^{2}\\ \Omega_{4}^{2} \end{array} \end{array}\right],$$

where *b* is the thrust coefficient, *l* is the distance from the center of mass to the respective motor, *d* is the drag coefficient, and $T_{\tau }$ is the control allocation matrix.

It is observed in (1) that all motors contribute to the torque around each axis of the aircraft. This is due to the quadcopter’s cross or X configuration, where no motor is aligned with a coordinate axis. The thrust force *u_z_* is equivalent to the sum of the forces generated by each propeller and is directed along *Z_b_* axis. Each of these forces equals the product of the thrust coefficient and the square of the respective motor’s angular velocity.

The quadcopter’s linear positions in space (*x*, *y*, *z*) are defined in the inertial coordinate system, as are its angular positions, which are associated with three Euler angles (*ϕ*, *θ*, and *ψ*—roll, pitch, and yaw, respectively), with the following limits: (–π/2 < *ϕ* < π/2), (–π/2 < *θ* < π/2), and (–π < *ψ* < π).

The dynamics of the quadcopter can be described by the Newton-Euler formulation, by initially considering that the aircraft’s structure is rigid, yielding (2).

(2) $$\left[\begin{array}{c} \ddot{x}\\ \ddot{y}\\ \ddot{z} \end{array}\right]=-g\left[\begin{array}{c} 0\\ 0\\ 1 \end{array}\right]+\frac{u_{z}}{m}\left[\begin{array}{c} C_{\psi }S_{\theta }C_{\Phi }+S_{\psi }S_{\Phi }\\ S_{\psi }S_{\theta }C_{\Phi }-C_{\psi }S_{\Phi }\\ C_{\theta }C_{\Phi } \end{array}\right],$$

where $\left(\ddot{x},\;\ddot{y},\;\ddot{z}\right)$ accounts for linear accelerations in the inertial coordinate system along the *x*, *y*, and *z* axes, respectively; *g* is the acceleration due to gravity; *m* is the mass; $C_{\Phi },C_{\theta },C_{\psi }$ represent the cosine operator applied to *ϕ*, *θ*, and *ψ*, respectively; $S_{\Phi },\;S_{\theta },\;S_{\psi }$ represent the sine operator applied to *ϕ*, *θ*, and *ψ*, respectively.

Thus, translational movement is described, where the linear accelerations in the inertial coordinate system are given as a function of the weight and the thrust force generated by the propellers. It is possible to describe the dynamic model of the quadcopter according to (3).

(3) $$\left[\begin{array}{c} \dot{p}\\ \dot{q}\\ \dot{r} \end{array}\right]=\left[\begin{array}{c} \frac{\left(I_{yy}-I_{zz}\right)qr}{I_{xx}}\\ \frac{\left(I_{zz}-I_{xx}\right)pr}{I_{yy}}\\ \frac{\left(I_{xx}-I_{yy}\right)pq}{I_{zz}} \end{array}\right]-I_{r}\left[\begin{array}{c} \frac{q}{I_{xx}}\\ -\frac{p}{I_{yy}}\\ 0 \end{array}\right]\left(\Omega_{1}-\Omega_{2}+\Omega_{3}-\Omega_{4}\right)+\left[\begin{array}{c} \frac{\tau_{\phi }}{I_{xx}}\\ \frac{\tau_{\theta }}{I_{yy}}\\ \frac{\tau_{\psi }}{I_{zz}} \end{array}\right],$$

where (*p*, *q*, *r*) is the angular velocity in the body-fixed reference frame, while $\left(\dot{p},\;\dot{q},\;\dot{r}\right)$ are the corresponding angular accelerations; *I_xx_*, *I_yy_*, and *I_zz_* are the moments of inertia about the *x*, *y*, and *z* axes, respectively.

Thus, the angular accelerations in the body-fixed coordinate system are described as functions of three torques: the torque associated with the Coriolis effect, the torque associated with the gyroscopic effect, and the torque produced by the propellers, respectively. To determine the angular accelerations in the inertial coordinate system, the Euler matrix is used, leading to (4), which describes the dynamic model of the quadcopter associated with the rotational system.

(4) $$\left[\begin{array}{c} \ddot{\Phi }\\ \ddot{\theta }\\ \ddot{\psi } \end{array}\right]=\ddot{\eta }=\left[\begin{array}{ccc} 0 & \dot{\phi }C_{\phi }T_{\theta }+\frac{\dot{\theta }S_{\phi }}{C_{\theta }} & -\dot{\phi }S_{\phi }C_{\theta }+\frac{\dot{\theta }C_{\phi }}{{C_{\theta }}^{2}}\\ 0 & -\dot{\phi }S_{\phi } & -\dot{\phi }C_{\phi }\\ 0 & \frac{\dot{\phi }C_{\phi }}{C_{\theta }}+\frac{\dot{\phi }S_{\phi }T_{\theta }}{C_{\theta }} & \frac{-\dot{\phi }S_{\phi }}{C_{\theta }}+\frac{\dot{\theta }C_{\phi }T_{\theta }}{C_{\theta }} \end{array}\right]\omega +W_{n}^{-1}\dot{\omega },$$

where $\left(\ddot{\phi },\ddot{\theta },\ddot{\psi }\right)$ is the angular acceleration in the inertial coordinate system and $\ddot{\eta }$ the corresponding vector; $T_{\Phi },\;T_{\theta },\;T_{\psi }$ represent the tangent operator applied to *ϕ*, *θ*, and *ψ*, respectively; $\dot{\phi }$ is the variation of the roll angle; ***W_n_*** is the Euler matrix; ω is the angular velocity vector in the body-fixed reference frame defined according to (3); and $\dot{\omega }$ is the corresponding angular acceleration. Thus, it is possible to define the nonlinear model of the quadcopter from (1), (2), and (4).

In this work, both the position control of the quadcopter and one of the filter approaches in the FDD structure relies on the system’s linear model. To obtain this model, hovering is considered as the operating point. Therefore, $\dot{\Phi }$, $\dot{\theta }$, and $\dot{\psi }$ are equal to zero, the angular velocities are the same for all motors, and *u_z_* is approximately equal to the quadcopter’s weight force. Additionally, to account for translational movement in the *x* and *y* directions, small roll and pitch angles are assumed, so that $\sin\left(\theta \right)$ and $\sin\left(\phi \right)$ are approximately equal to *θ* and *Φ*, respectively, while $\cos\left(\theta \right)$ and are equal to 1, respectively. This allows for deriving the quadcopter’s linear model, defined by (5).

(5) $$\left\{\begin{array}{l} \ddot{x}=\theta g\\ \ddot{y}=-\phi g\\ \ddot{z}=\frac{u_{z}}{m}-g\\ \ddot{\phi }=\frac{\tau_{\phi }}{I_{xx}}\\ \ddot{\theta }=\frac{\tau_{\theta }}{I_{yy}}\\ \ddot{\psi }=\frac{\tau_{\psi }}{I_{zz}} \end{array}\right..$$

## 3. Controller and FTC System Design

**Figure 2:**
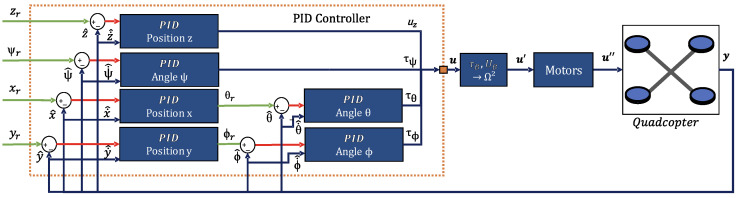
Initial configuration of the quadcopter’s control system.

A simple proportional-integral-derivative (PID) controller can be used to ensure that the quadcopter is in the desired position since this is a single input, single output (SISO) system [32]. Due to the decoupling between the linear model equations, a separate controller is required for each variable. In this work, a parallel PID controller with a derivative mode for measurement is adopted. The design method based on pole allocation is employed, as it calculates the parameter values by comparing the desired and actual system conditions. The gains are obtained directly from equations associated with the closed-loop transfer function and the initial configuration of the quadcopter’s control loop is shown in Figure 2.

Without an FTC and FDD architecture, the PID controller would be responsible for generating a control signal to counterbalance the losses caused by faults, with its performance depending on its response speed to the effects of the faults. Due to unexpected faults in the quadcopter’s propellers or motors, the actuators may exhibit undesirable behavior, raising concerns about reliability and safety. Faults associated with UAV actuators include high operating temperatures, heavy workloads on the motors, aging and wear of components (such as propellers and bearings), and electrical failures in the motor driver (short-circuit, open circuit, component aging, among others) [3].

In this work, actuator faults are considered, encompassing issues in all components from the propellers to the motors, represented by motor efficiency loss. The control signal is generated based on information from the sensors. Thus, the faulty control signal $u^{f}$ can be expressed in (6) as a function of the fault-free control signal u′ and the efficiency loss factor $\delta_{i}$ of the *i*-th motor [24].

(6) $$u_{i}^{f}=\left(1-\delta_{i}\right)u_{i}^{\prime },\;0\leq \delta_{i}\leq \delta_{i}^{max}\leq 1,$$

where $\delta_{i}^{max}$ represents the maximum allowable value of $\delta_{i}$ for the system to remain controllable. If the quadcopter operates correctly, then *δ_i_* = 0. A value of *δ_i_* > 0 indicates the presence of faults in the *i*-th actuator. Thus, it is possible to determine the control signal in the presence of faults from (7).

(7) $$u^{f}=u^{\prime }-U\delta =\left[\begin{array}{l} \Omega_{1}^{2}\\ \Omega_{2}^{2}\\ \Omega_{3}^{2}\\ \Omega_{4}^{2} \end{array}\right]-\left[\begin{array}{cccc} \Omega_{1}^{2} & 0 & 0 & 0\\ 0 & \Omega_{2}^{2} & 0 & 0\\ 0 & 0 & \Omega_{3}^{2} & 0\\ 0 & 0 & 0 & \Omega_{4}^{2} \end{array}\right]\;\left[\begin{array}{c} \delta_{1}\\ \delta_{2}\\ \delta_{3}\\ \delta_{4} \end{array}\right],$$

where U is the fault-free control signal matrix.

According to [33], environmental characteristics of the system and data that characterize sensor faults often include offset, noise, outliers, spikes, and freezing. Therefore, in this study, sensor faults will be considered as measurement faults. This behavior is described by the signal $f^{s}$ in (8), where $y^{f}$ is the generalized representation of a given faulty measurement signal ***y***.

(8) $$y^{f}=y+f^{s}.$$

### 3.1. Definitions for the Kalman Filter Algorithm

The initial system goal is to attenuate the measurement noise rather than estimate it. Outliers and spikes cause instantaneous variations with significant magnitude and correspond to a stage prior to fault estimations by the KF, which is an algorithm extensively used in various fields such as control, signal processing, and optimization. Due to its simple implementation, it has become popular in fault detection and diagnosis in stochastic processes, where the states are considered Gaussian random variables subject to zero-mean white noise [34]. This property allows for the definition of statistical characteristics for these variables, which will be described below as they are common parameters across all filter approaches.

Therefore, the following vectors are defined: the state vector of the quadcopter model ($x_{e}$ ∈ ℝ^n^), the system input signal vector (u′ ∈ ℝ^r^), the measurement vector (y ∈ ℝ^m^), the actuator fault vector ($f^{a}$ ∈ ℝ^p^), and the sensor fault vector ($f^{s}$ ∈ ℝ^q^) according to (9).

(9) $$\left\{\begin{array}{l} x_{e}={\left[x\;\;\;y\;\;\;z\;\;\;\dot{x}\;\;\;\dot{y}\;\;\;\dot{z}\;\;\;\Phi \;\;\;\theta \;\;\;\psi \;\;\;\dot{\Phi }\;\;\;\dot{\theta }\;\;\;\dot{\psi }\right]}^{T}\\ y={\left[x\;\;\;y\;\;\;z\;\;\;\dot{x}\;\;\;\dot{y}\;\;\;\dot{z}\;\;\;\Phi \;\;\;\theta \;\;\;\psi \;\;\;\dot{\Phi }\;\;\;\dot{\theta }\;\;\;\dot{\psi }\right]}^{T}\\ u^{\prime }={\left[\begin{array}{ccc} \Omega_{1}^{2} & \Omega_{2}^{2} & \begin{array}{cc} \Omega_{3}^{2} & \Omega_{4}^{2} \end{array} \end{array}\right]}^{T}\\ f^{a}={\left[\begin{array}{ccc} \delta_{1} & \delta_{2} & \begin{array}{cc} \delta_{3} & \delta_{4} \end{array} \end{array}\right]}^{T}\\ f^{s}={\left[f^{x}\;\;\;f^{y}\;\;\;f^{z}\;\;\;f^{\dot{x}}\;\;\;f^{\dot{y}}\;\;\;f^{\dot{z}}\;\;\;f^{\Phi }\;\;\;f^{\theta }\;\;\;f^{\psi }\;\;\;f^{\dot{\Phi }}\;\;\;f^{\dot{\theta }}\;\;\;f^{\dot{\psi }}\right]}^{T} \end{array}\right..$$

Thus, one can obtain the nonlinear and linear discrete models of the system corresponding to (10) and (11), respectively.

(10) $$\left\{\begin{array}{c} x_{e,k+1}=x_{e,k}+T_{s}f\left(x_{e,k},u_{k}^{\prime },f_{k}^{s}\right)+w_{k}^{x}\\ y_{k}=h\left(x_{e,k},u_{k}^{\prime },f_{k}^{s}\right)+v_{k}=C_{k}x_{e,k}+E_{k}f_{k}^{s}+v_{k} \end{array}\right.,$$

(11) $$\left\{\begin{array}{c} x_{e,k+1}=A_{k}x_{e,k}+B_{k}u_{k}^{\prime }+F_{k}f_{k}^{a}+w_{k}^{x}\;\\ y_{k}=C_{k}x_{e,k}+E_{k}f_{k}^{a}+v_{k} \end{array}\right.,$$

where $x_{e,k}$ and $x_{e,k+1}$ are the state vectors at the discrete time instants *k* and *k +* 1, respectively; *T_s_* is the sampling time; $w_{k}^{x}$ is the process noise vector; $v_{k}$ is the measurement noise vector; $y_{k}$ is the measurement vector at discrete time instant *k*; $A_{k},$ $B_{k}$, $C_{k},\;$$E_{k}$, and ***F_k_*** are matrices defined in (12), with ***I*** corresponding to the identity matrix; and ***A***, ***B***, ***C***, ***E***, and ***F*** are matrices obtained from the linear model of the quadrotor, sensors, and actuators fault according to (7) and (8).

(12) $$\left\{\begin{array}{l} A_{k}=I+T_{s}A\\ B_{k}=T_{s}B\\ C_{k}=C\\ E_{k}=E\\ \;F_{k}=T_{s}F \end{array}\right.\;.$$

To enable the KF to estimate faults in both actuators and sensors, these signals must be included in the state vector of the model, resulting in an augmented state space. In a previous work [35], the authors used a hybrid FDD system combining observers and neural networks for fault estimation. The system proved effective in estimating multiple faults, including simultaneous occurrences and nonlinear behaviors. Consequently, the following representations of the quadcopter are obtained in both linear and nonlinear augmented state spaces, with (13)–(19) incorporated into the model.

(13) $$\left\{\begin{array}{c} x_{e,k+1}^{a}=x_{e,k}^{a}+T_{s}f\left(x_{e,k}^{a},u_{k}^{\prime }\right)+w_{k}^{a}\\ y_{k}=h\left(x_{e,k}^{a},u_{k}^{\prime }\right)+v_{k}=C_{k}^{a}x_{e,k}^{a}+v_{k} \end{array}\right.,$$

(14) $$\left\{\begin{array}{c} x_{e,k+1}^{a}=A_{k}^{a}x_{e,k}^{a}+B_{k}^{a}u_{k}^{\prime }+w_{k}^{a}\;\\ y_{k}=C_{k}^{a}x_{e,k}^{a}+v_{k} \end{array}\right.,$$

where:

(15) $$A_{k}^{a}=\left[\begin{array}{ccc} A_{k} & F_{k} & 0\\ 0 & I & 0\\ 0 & 0 & I \end{array}\right],$$

(16) $$B_{k}^{a}=\left[\begin{array}{c} B_{k}\\ 0\\ 0 \end{array}\right],$$

(17) $$C_{k}^{a}=\left[\begin{array}{ccc} C_{k} & 0 & E_{k} \end{array}\right],$$

(18) $$x_{e,k+1}^{a}={\left[\begin{array}{ccc} {x_{e,k}}^{T} & {f_{k}^{a}}^{T} & {f_{k}^{s}}^{T} \end{array}\right]}^{T},$$

(19) $$w_{k}^{a}={\left[\begin{array}{ccc} {w_{k}^{x}}^{T} & {w_{k}^{fa}}^{T} & {w_{k}^{fs}}^{T} \end{array}\right]}^{T}.$$

A linear time-invariant system is considered observable if any initial state can be determined from the control input and the measurable output at any time greater than zero [36]. Observability can influence the KF’s ability to estimate states. For linear systems, observability is determined using the observability matrix ***O***, as shown in (20), assuming the aircraft is fault-free. The system is observable if the rank of this matrix equals the number of states.

(20) $$O=\left[{C_{k}^{a}}^{T}\;\;\;{A_{k}^{a}}^{T}{C_{k}^{a}}^{T}\;\;\ldots \;\;{\left({A_{k}^{a}}^{T}\right)}^{n+p+q-1}{C_{k}^{a}}^{T}\right].$$

It is observed that a loss of rank will occur in this case, rendering the system unobservable. Therefore, it is necessary to reduce the number of faults to be estimated. Faults in the actuators, being directly related to the control system, will remain unchanged. However, variables related to faults in linear and angular position measurements will be excluded from the sensor fault scenario. Thus, the new vector for sensor faults is defined by (21).

(21) $$f^{s}={\left[f^{\dot{x}}\;\;\;f^{\dot{y}}\;\;\;f^{\dot{z}}\;\;\;f^{\dot{\Phi }}\;\;\;f^{\dot{\theta }}\;\;\;f^{\dot{\psi }}\right]}^{T}.$$

Wind disturbances are considered to avoid false alarms in fault estimations. These disturbances affect the quadcopter by altering the system states according to their magnitude and direction. However, due to observability issues and the priority of estimating faults in actuators and sensors, these variables could not be included in the extended model of the filter.

### 3.2. KF Approaches

The filter algorithm is divided into two stages: prediction, which calculates the estimated value based on the system model, that is, the a priori value, denoted by the subscript *k*∣*k*−1; and update, where the a priori value is refined by the filter gain and measurements to yield the a posteriori value, denoted by *k*∣*k*.

This work considers three KF approaches: linear, EKF, and UKF. Additionally, the three-stage KF (TsKF), three-stage EKF (TsEKF), TsUKF, ATsKF, adaptive three-stage EKF (ATsEKF), and adaptive three-stage UKF (ATsUKF) are assessed, totaling nine solutions. Except for the adaptive structure of the ATsUKF, the KF approaches were implemented according to guidelines provided in the studies mentioned in Table 2.

One issue with the KF is the presence of model nonlinearities, which lead to different behaviors of the states compared to the linear model, especially in the presence of unknown dynamics, such as faults [25]. To mitigate these harmful effects, the EKF uses the nonlinear model in the prediction step and performs linearizations of the model based on the current values of each state at each sampling period during the update step [37]. However, these linearizations can introduce errors in the update process. For this reason, the UKF is used to address this issue by propagating sigma points through the nonlinear system model [36].

The drawback of using more complex KF structures, such as the EKF and UKF, and especially system models with an expanded state space to include actuator and sensor faults, is the considerable increase in computational burden. One way to minimize it consists of dividing the estimations into three stages, creating three subfilters, each related to the states of the quadcopter’s flight dynamics, actuator faults, and sensor faults. The subfilters are divided by applying the U-V transformation to the covariance matrix, state vector, and gain vector of the respective filter [25,38]. After each subfilter estimates its states, there is a correction step, characterized by incorporating the sensor fault estimations into the actuator fault estimations and then integrating these into the estimations of the quadcopter’s flight states.

It is known that actuator and sensor faults have unknown dynamics, and for this reason, there will be a deterioration in the filter’s performance during the estimation process. To solve this problem, there are adaptive versions that adjust the filter parameters based on estimations over time. In this work, the adaptive approach implemented in the TsUKF filter described in [25] is based on the forgetting factor *λ* for weighting the covariance matrix $P_{k|k-1}$ defined in (22) during the prediction step at time instant *k*. The matrix $P_{k|k-1}^{\lambda }$ corresponds to the covariance matrix for calculating the filter gain and other variables.

(22) $$P_{k|k-1}^{\lambda }=\lambda P_{k|k-1}.$$

The forgetting factor *λ* can be determined from (23) and (24).

(23) $${\tilde{P}}_{vv,k}=\frac{1}{M-1}\sum_{i=k-M+1}^{k}v_{k}v_{k}^{t},$$

(24) $$\lambda =\max\left\{1,\;\frac{tr\left({\tilde{P}}_{vv,k}\right)}{P_{vv,k}}\right\},$$

where *v_k_* is the innovation, *M* is the window that defines the number of samples for calculating the covariance ${\tilde{P}}_{vv,k}$, and $P_{vv,k}$ is the covariance of the innovation calculated analytically, which corresponds to the term whose inverse is calculated in the filter gain equation.

The innovation covariance matrix is used in (23) because the filter will be optimal in the context of the mean squared error if the model is accurate and both matrices are equal [24]. Furthermore, the ability to calculate ${\tilde{P}}_{vv,k}$ in real-time makes such matrices attractive. However, due to the presence of model errors and the unknown nature of certain statistical properties of the signals, these values may differ, representing a degradation of the optimal value of the filter gain. Therefore, it can be noted in (23) that if the values of the analytically calculated matrix are less than those of the other matrix, *λ* will be greater than one, consequently increasing the values of the covariance matrix and the filter gain. The higher the gain, the greater the relevance of the innovation in updating the estimations compared to the prediction. In the context of model uncertainties, this allows for an adjustment in the convergence of the filter’s covariance matrix based on the most current values obtained from the system, reducing estimation errors. Thus, the forgetting factor is used to mitigate potential undesirable effects of old information generated by model issues.

Additionally, the presence of “1” in (23) prevents the covariance matrix from being smaller than that obtained by the filter analytically, as this would produce the opposite effect to what was previously discussed, degrading the estimation performance. It is important to note that for three-stage filters, each sub-filter will have its innovation, and thus each element must calculate its respective forgetting factor.

### 3.3. FTC Structure

FTC systems are designed to mitigate or neutralize the effects of faults in the control loop to ensure good performance and stability of the process. The FTC method introduced in this work relies on estimations from the FDD method, functioning similarly to feedforward control, as described by (25).

(25) $$u_{k}^{m}=u_{k}^{\prime }+U_{k}^{m}{\hat{f}}_{k}^{a},$$

where $u_{k}^{\prime }$ is the control signal vector generated by the PID and converted into a signal suitable for the motors at time *k*, ${\hat{f}}_{k}^{a}$ is the vector of actuator faults estimated by the FDD system, $U_{k}^{m}$ is the matrix related to the angular velocities of the motors, and $u_{k}^{m}$ is the final control signal transmitted to the quadcopter’s actuators.

By using the representation of actuator faults as described in (6) and converting the signals to another suitable signal for the motors, it is possible to obtain (26).

(26) $$u_{k}^{m\prime }=u_{k}^{m}-U_{k}^{m}f_{k}^{a},$$

where $u_{k}^{m\prime }$ is the control signal with the presence of actuator faults. As discussed earlier, due to these faults, this control signal will not have the same value generated by the PID controller without the FTC system, which would lead to a degradation of the drone’s flight. However, substituting (25) in (26) yields a control signal that compensates for the effects of actuator faults in the system, eliminating this problem through the FTC, corresponding to (27). It is observed that the smaller the error between the actual value of the fault and the estimated value, the better the compensation will be.

(27) $$u_{k}^{m\prime }=u_{k}^{\prime }+U_{k}^{m}{\hat{f}}_{k}^{a}-U_{k}^{m}f_{k}^{a}=u_{k}^{\prime }.$$

**Figure 3:**
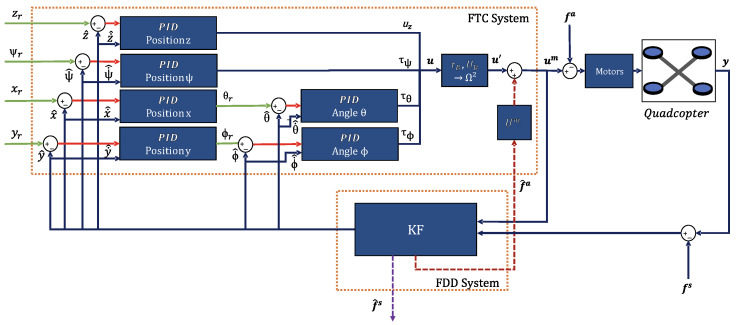
FDD and FTC systems implemented for the quadcopter.

For sensor faults, a control loop based solely on PID controllers would be unable to distinguish between accurate measurements and those distorted by faults. Consequently, such a system would consistently misposition the quadcopter. By using FDD and FTC systems, the feedback in the PID controllers occurs using only the values of the flight dynamics states, which are decoupled from the sensor faults by the Kalman filter. Therefore, this approach allows for avoiding the undesirable problem discussed earlier. The complete system, involving FDD, FTC, the PID controller, and the quadcopter, is shown in Figure 3.

## 4. Results and Discussion

In this work, the Parrot Minidrone, provided by the MathWorks Simulink Team since 2017, is used as the quadcopter model for implementing FDD and FTC systems. This aircraft is equipped with an ultrasonic sensor, accelerometer, gyroscope, air pressure sensor, and front camera, which together enable the measurement of its linear and angular positions and velocities in both coordinate systems, either directly or indirectly via numerical integrations and transformation matrices.

To assess the filtering performance, the integral absolute error (IAE) metric was applied to the actual and estimated values, and these results were normalized for better comparison of performance. Table 3 shows the calculated PID controller gains for the controlled variable, where *OS* stands for the maximum overshoot; *T_s_*_,5%_ is the settling time for a tolerance band of 5%; *K_p_*, *K_i_*, and *K_d_* are the proportional gain, integral gain, and derivative gain, respectively. The sampling time is 5 ms and lower settling times were chosen for the roll and pitch angles compared to the other angles due to the cascade structure with the *x* and *y* position controllers. Conversely, Table 4 presents the parameters for all the KF approaches used in the simulations.

The noise covariance matrices, particularly those associated with faults, were manually adjusted to ensure satisfactory convergence speed, reduce overshoot in estimations, and attenuate measurement noise. These three factors are directly associated with the performance of the FTC method in mitigating the effects of faults on the system, which can be degraded by delays in detecting the actual fault value or by the incorrect insertion of information into the control signal. The measurement noise covariance matrix was obtained along with other quadcopter parameters.

The results from the implementation of the FDD and FTC methods on the quadcopter were obtained through simulations in Simulink. For analysis, the integral absolute error (IAE) metric was employed for the actual and estimated values, with all values normalized. The simulations considered two flight trajectories: an initial ascending movement followed by a rectangular movement in the XY plane while maintaining a constant vertical position. The complete movement of the quadcopter is performed in 35 s.

One of the advantages of using FDD methods based on the Kalman filter is the ability to estimate faults in actuators and sensors with nonlinear behaviors and multiple occurrences. To address this scenario, faults were introduced into each of these quadcopter elements according to Table 5. The magnitude is based on the percentage of the nominal value of the variable affected by the respective fault. For actuator faults, the value was determined based on the parameter *δ*_max_, which corresponds to the maximum allowable fault value that ensures system stability. The sensor faults were introduced into the system as a step change to represent offset. Separate simulations will be conducted for scenarios involving lockups. So, the offset was chosen to account for various measurement situations.

### 4.1. Scenario 1: ATsUKF

**Figure 4:**
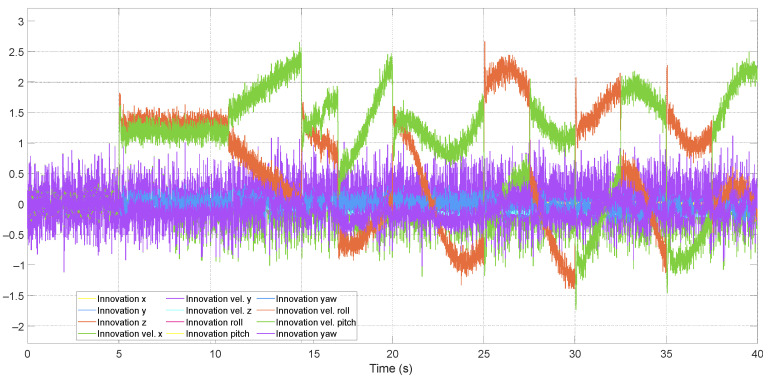
Innovation of the fault sub-filter using ATsUKF.

Figure 4 compares two adaptive structures by weighting the innovation covariance matrix: one across all three sub-filters (structure 1) and the other only in the sensor fault sub-filter (structure 2). Structure 1 exhibited some sensitivity to variations in the time interval *M* and a deterioration in estimations compared to structure 2.

It is observed that using the forgetting factor in all three sub-filters results in a deterioration in estimation compared to using it in just one sub-filter. Additionally, structure 2 is more sensitive to variations in the time interval determined by *M*. In turn, structure 1 proved to be almost insensitive to this variation. This behavior is also observed in the ATsKF and ATsEKF filters. The explanation for this behavior lies in the averages of the innovations of the state and actuator fault sub-filters, which will not be zero in the presence of a second signal in the system. This occurs due to the correction of estimates at the end of the algorithm. Consequently, large values of *M* will affect the covariance calculation if the innovation exhibits non-stationary and persistent behaviors over time, such as sinusoidal and intermittent signals, while small values of *M* will be sensitive to sudden variations in the signal.

### 4.2. Scenario 2: Performance of Different FDD Approaches

This analysis focuses on the FDD system’s ability to estimate all faults simultaneously, highlighting its accuracy and the isolation of estimations. This latter factor is associated with the system’s ability to diagnose a fault without significant and persistent interference from other faults. Table 6 summarizes the filtering performance in this scenario using the normalized IAE value for each variable.

**Figure 5:**
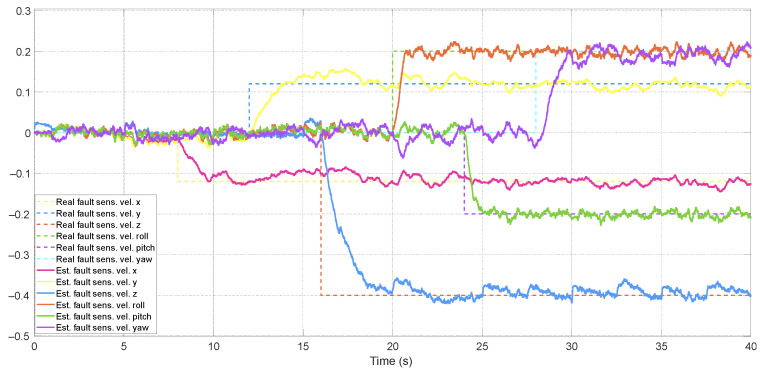
Estimation of sensor faults using ATsUKF.

**Figure 6:**
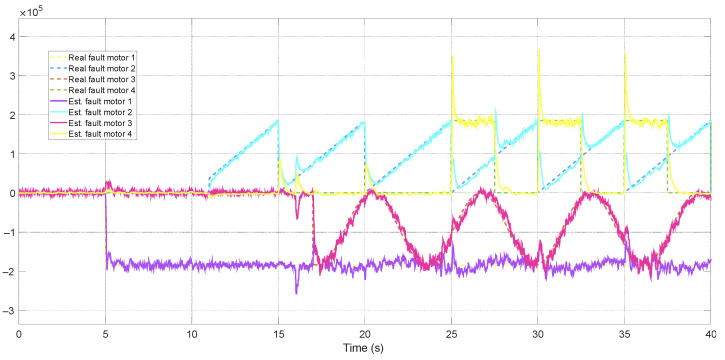
Estimation of actuator faults using ATsUKF.

The adaptive approach improved estimation accuracy by up to 30%. Due to the number of filters and the simulation time interval, which makes it challenging to observe performance differences over a short period, only the faults estimated by the ATsUKF are illustrated in Figure 5 and Figure 6. Figure 6 shows that one estimated actuator fault is characterized by peaks generated by the variation of another fault, with a duration of less than approximately 0.5 s. The magnitude of these peaks is directly associated with the convergence speed of the estimation. The filter can estimate faults through innovation, which depends on the measurement signals, and these are related to the states of the aircraft dynamics. According to the equations representing the quadcopter model, all actuator faults affect a state variable. Therefore, transients are a consequence of the interval required by the filter to correctly determine the fault’s location. Since these signals influence the control signal in the FTC architecture, they can induce undesirable behavior in the quadcopter, which may be partially interpreted as measurement problems.

By comparing the performance of linear (KF) and nonlinear (EKF and UKF) filters, it is observed that both exhibited similar behavior in estimating the states of the quadcopter. However, the nonlinear versions achieved a maximum reduction of 7% in the IAE of actuator faults and a maximum reduction of 48% in the IAE of sensor faults.

EKF and UKF showed similar performance with a maximum difference of 1%, except for some faults, where the difference reached 4% and 11%, with the UKF having the worst performance. The three-stage version performs linearization based on faults, and the propagation of sigma points is limited to the quadcopter’s dynamic states. It is observed that TsUFK shows improved estimations compared to the UKF and similar results to the EKF, reducing all differences to less than 0.5%. This demonstrates that, for the nonlinear dynamics considered and the movement of the quadcopter, the second moment of the Taylor series does not have a significant effect on the precision of the mean and covariance, and does not necessarily benefit estimation in cases involving faults with unknown models.

As for the adaptive filters, there was an increase in accuracy of 22% to 30% for $f^{\dot{z}}$ and 7% to 13% for $f^{\dot{\psi }}$ when compared to the same types of filters without the adaptive structure. For the other variables, the maximum improvement was 7%, with an average of around 2%.

### 4.3. Scenario 3: Performance of the FTC Architecture

**Figure 7:**
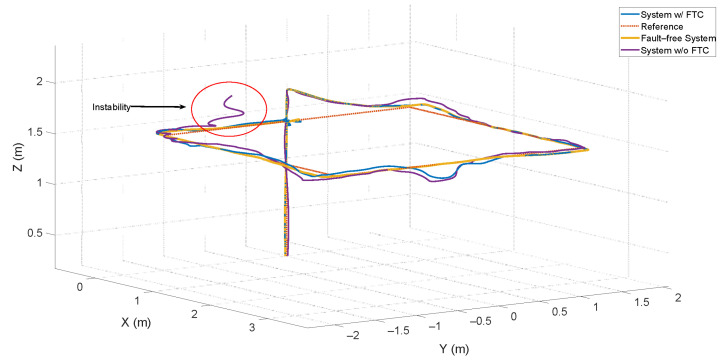
Displacement of the quadcopter subjected to actuator and sensor faults.

The third scenario relies on the ATsUKF, highlighting the advantage of using an FDD method to anticipate faults and reduce the controller’s effort in mitigating the effects of these signals on the system. The results for the *x*, *y*, and *z* positions are shown in Figure 7.

The main goal of the FTC is to mitigate the effects of faults in the process and ensure stability. Figure 7 shows that, without this structure, the system becomes unstable at a certain instant, with oscillations around the fault-free system response. The PID controller alone responds to faults, but since it relies solely on the feedback error, the angular velocities, and the linear velocities, its response is slower compared to systems based on filters like FDD. This is because FDD systems use a model of the aircraft’s behavior considering different types of faults.

**Figure 8:**
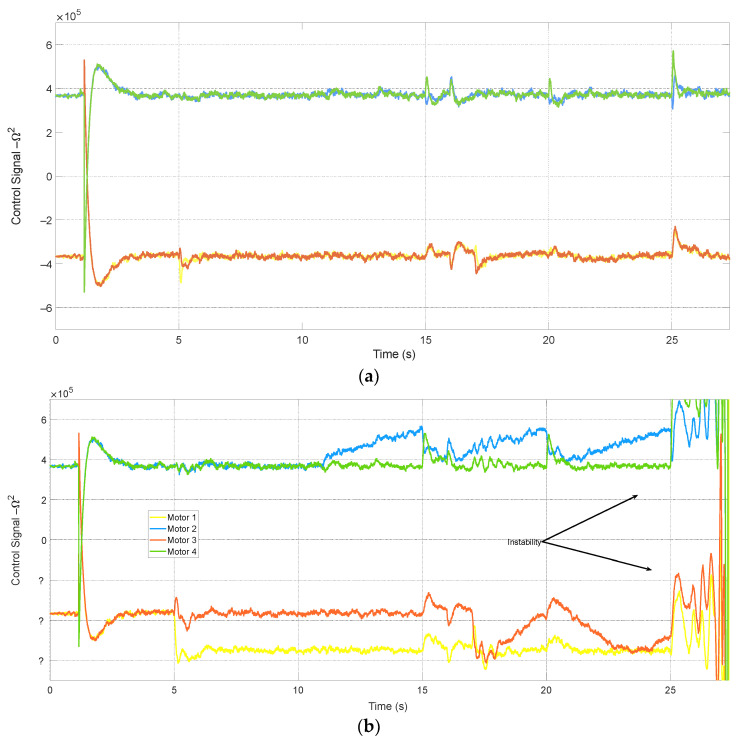
Control signals generated in systems with (**a**) and without (**b**) FTC.

Figure 8 compares the signals generated by the PID controllers in systems with and without FTC. It shows that all fault attenuation actions are managed by the FDD system, while the short-term variations in the PID controller of the FTC system are related to estimation transients. Additionally, without FTC, the measurement noise was reduced using a KF that only considers the quadcopter model.

**Figure 9:**
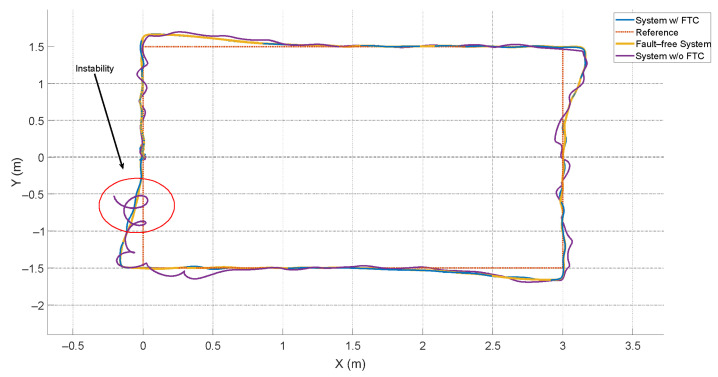
Quadcopter displacement in the *xy* plane.

Figure 9 shows the quadcopter’s displacement in the XY plane. It demonstrates that the system with FTC achieved oscillations with a maximum value of less than 2%, partly due to reduced interference from transients in fault estimation for velocity measurements in the *x* and *y* directions, as previously discussed.

### 4.4. Scenario 4: Performance of the FDD and FTC Architectures with Lock-Up Faults

**Figure 10:**
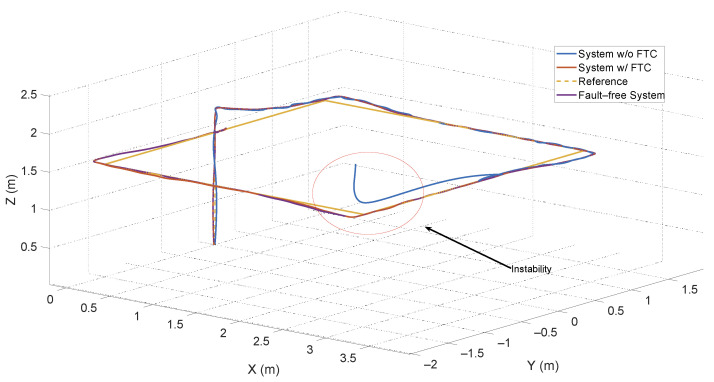
Behavior of systems in the presence of lock-up sensor faults.

The fourth scenario comprises lock-up faults, where the measurement signal remains constant at the last value before the fault occurs for some time, demonstrating the efficiency of the FDD structure. Figure 10 shows the behavior of different systems under these conditions. The fault was introduced in the *y*-axis velocity measurement at 14 s, maintaining the measurement value fixed at −0.6 m/s until 24 s into the simulation, at which point the real values reappeared. All other faults were removed from the system to observe the isolated behavior of the lock-up fault.

The system without FTC became unstable 4.0 s after the onset of the lock-up fault. In this case, controllers are more vulnerable compared to faults in actuators and offset behaviors in measurements. The disturbance caused by the lock-up fault leads the integral mode to continuously act increasingly since the measurement is locked at a value, making the feedback error constant and non-zero. This results in process instability if the measurement does not return to its normal operating value.

**Figure 11:**
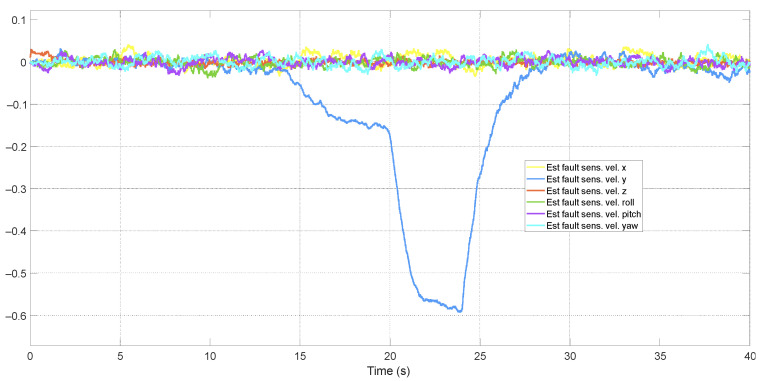
Estimation of sensor lock-up faults.

Figure 11 shows the estimation of this fault by the KF, corresponding to the difference between the lock-up value and the actual target variable value. The FDD system can distinguish between the real signal, which is sent to the controller, and the faulty signal caused by the lock-up, preventing the same issues in the controller as seen in systems without this structure, thereby maintaining aircraft stability.

### 4.5. Scenario 5: Performance of the FDD and FTC Architectures with Disturbances

The fifth scenario consists of assessing the influence of external disturbances, specifically wind, on the fault estimation process. Winds alter the system’s state values based on their direction and magnitude. This behavior can be described by proper equations as presented in [39], which uses the Beaufort scale to determine wind speed according to the environment.

**Figure 12:**
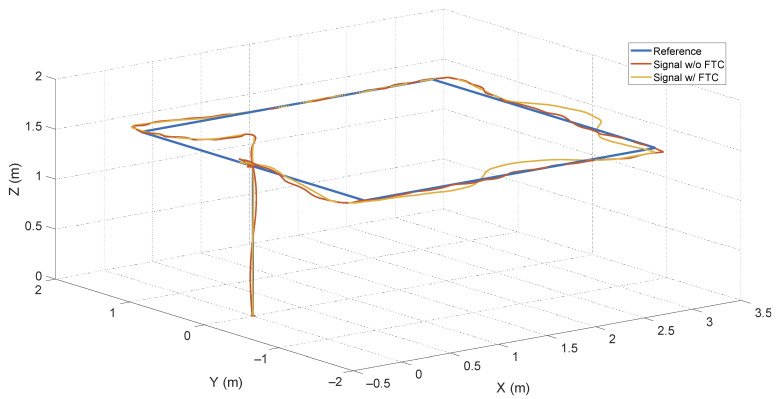
Behavior of systems in the presence of wind-generated disturbances.

Thus, the impact of these disturbances is analyzed since they were not considered in the quadcopter model. The simulation represents this external signal in a generalized manner as a constant non-null value for some time. Figure 12 shows the system’s response to the following disturbances: forces applied in the *x*, *y*, and *z* directions at 15 s, 21 s, and 27 s, respectively, with a constant value *u_z_* = 10% and a duration of 2s. The FTC system’s response to disturbances has deteriorated compared to the system without FTC because false alarms are generated by fault estimations in the sensors and actuators.

**Figure 13:**
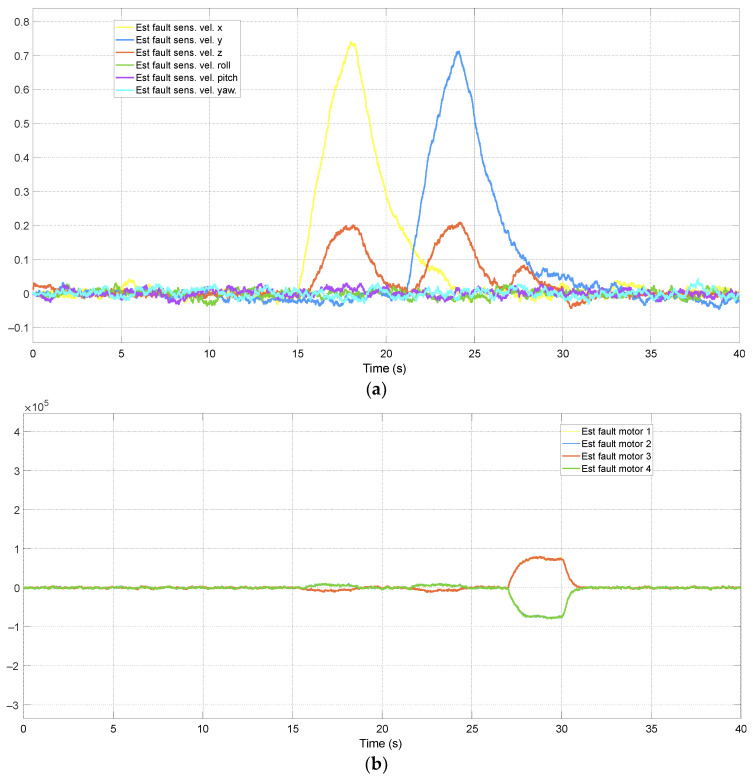
Fault estimations in (**a**) sensors and (**b**) actuators.

According to Figure 13, these false alarms inject erroneous signals into the control loop. Consequently, they force the controller—the only component capable of mitigating disturbances—to act in a way that reduces the effects of wind along with those generated by the filter. For the system without FTC, the PID only addresses the wind effects.

It is also important to note that winds are not fixed disturbances, as they vary with various environmental factors. Thus, false alarms are similar to transients that cancel out after a while. For this reason, depending on the duration, magnitude, and direction of the wind (given that the disturbance in the *z* direction had a considerably smaller effect compared to *x* and *y*), the FTC system may not experience significant performance degradation.

## 5. Conclusions

The combined use of FDD and FTC has improved the reliability and safety factors of the aircraft in terms of stability and flight performance in fault situations, compared to systems without FTC. From the results obtained, by comparing different FDD structures based on the KF and the performance of fault-tolerant controllers, the following issues were observed:

- transients in estimations generate disturbances in the control loop;
- adaptive approaches enhance estimation accuracy;
- nonlinear filters perform better than linear ones;
- FTC systems effectively maintain quadcopter stability in the presence of faults;
- FDD and FTC methods present some drawbacks when dealing with external disturbances.

FDD structures were able to accurately estimate the considered faults. In the case of model-based FDD methods, the efficiency is directly influenced by the amount of information available about the process. Transients in estimation represent false fault alarms, and in FTC systems, these can introduce disturbances in the control loop, affecting the quadcopter’s performance when wind-related disturbances exist. However, the effectiveness of the FTC system in maintaining stability during actuator and measurement faults, particularly those with lock-up behavior, was confirmed. Additionally, it is important to consider the fault signal frequency when designing Kalman filters due to their convergence speed. The adaptive approach positively contributed to selecting an estimator that significantly reduces measurement noise while providing the desired estimation speed.

Future work includes applying FDD and FTC methods to real UAVs. If testing with defective components is not feasible due to difficulty in obtaining faults experimentally, there may be a possibility of simulating these faults computationally. Another area of interest is studying the performance of FDD and FTC systems under different flight conditions, including larger variations in roll, pitch, and yaw angles, to assess the impact of nonlinearities. Exploring other control strategies, such as model predictive controllers and robust controllers, is also possible.

## Figures and Tables

**Table 1:** Comparison of FDD and FTC methods.

Solutions	Characteristics
Signal-based methods	- Focus on fault detection for maintenance purposes only, without enabling diagnosis; - Additional sensors may be required; - Least recommended solution for the specific case of this work.
Data-driven methods	- Diagnosis aimed at classifying faults, except in hybrid cases; - Historical fault data are required; - Application in artificial intelligence (AI) faces challenges due to the difficulty of knowing all possible faults.
Model-based methods	- Diagnosis based on fault estimation, with the capacity to isolate and determine the magnitude, location, and time of occurrence; - Performance is affected by the model accuracy; - Mostly recommended for drones, as used in this work.
All methods	- Problems in integrating sensor and actuator faults into the FTC; - Lack of results related to certain scenarios, such as simultaneous and multiple occurrences of faults and the presence of disturbances in the system.

**Table 2:** Comparison of KF-based algorithms.

Version	Linear	Nonlinear	Application
Enhanced	KF	UKF [36] EKF [37]	- Incorporation of actuator and sensor faults into the system’s state space for estimating these signals. - Performance assessment of both versions in the presence of nonlinearities and unknown fault dynamics.
Three-Stage	TsKF	TsUKF [25] TsEKF [38]	- Possible incorporation of three sub-filters, related to the quadcopter dynamics and faults in actuators and sensors. - Reduced computational burden.
Adaptive	ATsKF	ATsEKF [24], ATsUKF	- The actual innovation covariance is weighted against the innovation covariance obtained analytically from the filter. - Improved accuracy in fault estimation.

**Table 3:** Parameters of the PID controller.

Controlled Variable	*OS* (%)	*T_s,_*_5*%*_ (s)	Third Pole	*K_p_*	*K_i_*	*K_d_*
*x*	<1	2	6	2.11	1.66	9.17·10^−1^
*y*	<1	2	6	−2.11	−1.66	−9.17·10^−1^
*z*	<1	2	6	1.31	1.03	0.57·10^−1^
*Φ*	<1	0.5	24	1.93·10^−2^	6.08·10^−2^	2.10·10^−2^
*θ*	<1	0.5	24	2.38·10^−2^	7.48·10^−2^	2.58·10^−2^
*ψ*	<1	2	6	2.10·10^−3^	2.58·10^−3^	9.00·10^−4^

**Table 4:** Parameters of the KF-based algorithms.

Parameter	Description
$Q^{x}$	Diagonal matrix: 2.5·[10^−6^ 10^−6^ 10^−6^ 10^−6^ 10^−6^ 10^−6^ 10^−8^ 10^−8^ 10^−8^ 10^−8^ 10^−8^ 10^−8^]
$Q^{fa}$	Diagonal matrix: 3.0·[10^−6^ 10^−6^ 10^−6^ 10^−6^]
$Q^{fs}$	Diagonal matrix: 3.0·[10^−6^ 10^−6^ 10^−6^ 10^−6^ 10^−6^ 10^−6^]
$Q^{xfa},Q^{xfs},Q^{fafs}$	Null matrices
$P_{0}^{x}$	Diagonal matrix: [10^−4^ 10^−4^ 10^−4^ 10^−4^ 10^−4^ 10^−4^ 10^−4^ 10^−4^ 10^−4^ 10^−4^ 10^−4^ 10^−4^]
$P_{0}^{fa}$	Diagonal matrix: [10^−4^ 10^−4^ 10^−4^ 10^−4^];
$P_{0}^{fs}$	Diagonal matrix: [10^−4^ 10^−4^ 10^−4^ 10^−4^ 10^−4^ 10^−4^]
$P_{0}^{xfa},P_{0}^{xfs},P_{0}^{fafs}$	Null matrices
R	Diagonal matrix: [10^−3^ 10^−3^ 5.0·10^−4^ 10^−1^ 10^−1^ 5.0·10^−4^ 10^−4^ 10^−4^ 10^−2^ 10^−2^ 10^−2^ 10^−2^]
*M*	50 samples

**Table 5:** Fault scenarios.

Fault	Instant (s)	Behavior	Maximum Amplitude (%)
$\delta_{1}$	5	Step	50
$\delta_{2}$	11	Sawtooth	50
$\delta_{3}$	17	Sinusoidal	50
$\delta_{4}$	23	Inttermitent	50
$f^{\dot{x}}$	8	Step	20
$f^{\dot{y}}$	12	Step	20
$f^{\dot{z}}$	16	Step	20
$f^{\dot{\Phi }}$	20	Step	15
$f^{\dot{\theta }}$	24	Step	15
$f^{\dot{\psi }}$	28	Step	40

**Table 6:** Performance of the filters under actuator and sensor faults.

Variable	KF	EKF	UKF	TsKF	TsEKF	TsUKF	ATsKF	ATsEKF	ATsUKF
x	0.998	0.992	0.992	0.998	0.992	0.992	1.000	0.994	0.994
y	0.999	0.998	1.000	0.999	0.998	0.998	1.000	0.999	0.999
z	0.985	0.992	1.000	0.985	0.992	0.992	0.951	0.951	0.951
$\dot{x}$	1.000	0.974	0.974	1.000	0.974	0.974	0.990	0.968	0.968
$\dot{y}$	0.992	0.981	0.981	0.992	0.981	0.981	1.000	0.987	0.987
$\dot{z}$	0.976	0.973	0.969	0.976	0.973	0.973	1.000	1.000	1.000
Φ	0.956	0.959	1.000	0.956	0.959	0.959	0.957	0.957	0.957
θ	0.985	0.980	1.000	0.985	0.980	0.980	0.980	0.976	0.976
ψ	0.975	1.000	0.990	0.975	1.000	1.000	0.932	0.926	0.926
$\dot{\Phi }$	0.998	1.000	0.998	0.998	1.000	1.000	0.994	0.996	0.997
$\dot{\theta }$	0.998	1.000	0.997	0.998	1.000	1.000	0.997	1.000	1.000
$\dot{\psi }$	0.995	0.953	0.958	0.995	0.953	0.953	1.000	0.953	0.953
$f^{\dot{x}}$	1.000	0.933	0.938	1.000	0.933	0.933	0.990	0.931	0.931
$f^{\dot{y}}$	1.000	0.976	0.973	1.000	0.976	0.976	0.963	0.946	0.946
$f^{\dot{z}}$	0.924	0.965	1.000	0.924	0.965	0.965	0.695	0.709	0.709
$f^{\dot{\Phi }}$	0.893	0.897	1.000	0.893	0.897	0.897	0.882	0.885	0.885
$f^{\dot{\theta }}$	0.948	0.948	1.000	0.948	0.948	0.948	0.940	0.938	0.938
$f^{\dot{\psi }}$	0.903	1.000	0.979	0.903	1.000	1.000	0.830	0.871	0.871
$\delta_{1}$	0.990	0.582	0.579	0.990	0.582	0.582	1.000	0.583	0.583
$\delta_{2}$	0.985	0.507	0.500	0.985	0.507	0.509	1.000	0.503	0.505
$\delta_{3}$	0.991	0.598	0.600	0.991	0.598	0.597	1.000	0.600	0.600
$\delta_{4}$	0.985	0.461	0.452	0.985	0.461	0.462	1.000	0.458	0.459

## Data Availability

Data are available upon request from the authors.
